# Association of teen mothers’ and grandmothers’ parenting capacities with child development: A study protocol

**DOI:** 10.1002/nur.21839

**Published:** 2017-11-11

**Authors:** Damali Wilson, Deborah Gross, Stacy Hodgkinson, Kirby Deater‐Deckard

**Affiliations:** ^1^ Johns Hopkins University School of Nursing Baltimore Maryland; ^2^ Generations Program, Children's National Health System Washington, DC; ^3^ University of Massachusetts Amherst Amherst Massachusetts

**Keywords:** early child development, executive function and parenting, intergenerational parenting, teen mothers

## Abstract

Children born to teen mothers may experience less responsive and supportive parenting and are at heightened risk for a range of social, developmental, and health issues. There is literature to support the positive impact of grandmothers on teen parents and their children. However, what if the teen's mother is also limited in her parenting capacities? How do parenting capacities across these two generations of mothers affect the developing child? In this ongoing study we are examining two important aspects of parenting capacities, attachment quality and executive functioning, in teen mothers (TM) and their biological, co‐ residing mothers or grandmothers (GM or GGM). Both are essential components of effective parenting, but little is known about their impact on young children's development when raised by two generations of parents. In a cross‐ sectional, descriptive design, a convenience sample of 50 TM/GM dyads with children 1 to 3 years old is being recruited from two urban teen‐tot clinics. Participants complete a paper‐and‐pencil measure of attachment quality and a computerized measure of multiple aspects of executive function (working memory, inhibitory control, cognitive flexibility). A standardized maternal report measure is used to assess child developmental status. The biggest challenges of the study thus far include recruitment and transience of the study population. Progress to date and experiences from recruitment and data collection are discussed, as well as successful strategies to address challenges.

## 1 INTRODUCTION

Teen birthrates in the United States have declined over the last 20 years to historic lows, but disproportionately higher rates persist among Blacks and Latinos (National Campaign to Prevent Teen and Unplanned Pregnancy, 2016). The negative sequelae of this phenomenon can be substantial. Teen mothers in general are less interactive, less positive in their parenting style, and have more difficulties with problem solving and harsher parenting behavior (Beers & Hollo, 2009; Lee & Guterman, 2010). Children born to teen mothers are at heightened risk for developmental delays, deficits in cognitive and social development, and behavioral problems (Mollborn & Dennis, 2012; Pinzon, Jones, & Committee on Adolescence & Committee on Early Childhood, 2012; Ruedingera & Cox, 2012).

While several parenting program models exist for teen mothers (Olds et al., 2007), few have been rigorously evaluated or shown to be effective for improving outcomes among teen parents and their children (Lachance, Burrus, & Scott, 2012). To better understand aspects of teen parenting that most need to be targeted to improve parenting quality and child outcomes in this high‐risk population, the purpose of this ongoing study is to better understand the association of the attachment and executive function capacities of teen mothers and their mothers (grandmothers of the child) or grandmothers (great‐grandmothers of the child) with the development of their young children.

## 2 BACKGROUND

The association between parenting quality and young children's cognitive, language, social‐emotional, and behavioral development is well established (Barnett, Gustafsson, Deng, Mills‐Koonce, & Cox, 2012; Ermisch, 2008; Glascoe & Leew, 2010). Parenting requires the ability to form strong emotional connections to others (attachment quality) as well as the ability to organize, plan, and exhibit self‐control (aspects of executive functioning). Parents with secure attachment styles tend to be more responsive, sensitive and involved, leading to a secure foundation from which a young child explores, learns, and develops (Ainsworth, 1979; Bowlby, 1982). Nurturing care, including secure attachment, is a contributor to early childhood cognitive and psychosocial development (Britto et al., 2017). In contrast, an avoidant pattern of attachment has been linked to less sensitive and responsive parenting, discomfort with close relationships, and greater likelihood of missing cues that the child needs care and support (Berlin et al., 2011; Edelstein et al., 2004; Selcuk et al., 2010). There is evidence that adolescent mothers exhibit more insecure attachment styles than do adult mothers (Crugnola, Ierardi, Gazzotti, & Albizzati, 2014; Lewin, Mitchell, & Ronzio, 2013). In a young, economically disadvantaged sample of mothers, a large and significant relationship (*r* = −.84) was found between mothers’ avoidant attachment style and risk for developmental delay in their 1 to 2 year old children (Alhusen, Hayat, & Gross, 2013).

Although much is known about attachment in parent‐infant dyads, less is known about grandparents and the parent‐grandparent dyad. Further, while significant attachment is documented between grandmothers and mothers and between young adults and their parents, these findings have been among middle‐ and high‐income, mostly two‐parent White households (Benoit & Parker, 1994; Cassibba, Coppola, Sette, Curci, & Costantini, 2017; Hautamäki, Hautamäki, Neuvonen, & Piispanen, 2010). These attachment associations have not been fully explored among other populations. Because many young mothers are raising their young children in tandem with their own mothers, the nature of their attachment relationships is important to understand.

High‐quality parenting (interactive, responsive, supportive, sensitive parenting) also requires that parents have certain cognitive capacities or executive function (EF) skills. These skills include directing one's attention; flexibility in shifting attention as circumstances and environments change; generating responses to a child's needs and behaviors; planning, prioritizing, retaining, and applying information about a child; and problem‐solving (Azar, Reitz, & Goslin, 2008; Azar & Weinzierl, 2005; Barrett & Fleming, 2011). There is a significant association between parental EF and parenting behaviors (Deater‐Deckard & Sturge‐Apple, 2017). In particular, low parental EF is linked to more negative parenting behaviors (reactivity; rigid, harsh discipline; and child maltreatment), and higher EF is linked to more positive parenting practices (supportive responses to child's emotions, maternal warmth and sensitivity; Crandall, Deater‐Deckard, & Riley, 2015; Deater‐Deckard, Sewell, Petrill, & Thompson, 2010; Deater‐Deckard, Wang, Chen, & Bell, 2012; Valiente, Lemery‐Chalfant, & Reiser, 2007). Further, maternal EF has been shown to significantly influence child EF (Cuevas et al., 2014; De Cock et al., 2017). While EF is interrelated with cognitive and social development in early childhood (Blasco, Saxton, & Gerrie, 2013; Center on the Developing Child at Harvard University, 2011), the associations between maternal EF and their children's broad range of developmental outcomes is less well understood.

Of the teen mothers who had given birth in 2008–2010, 72% were living with their parent or other adult relative (Ng & Kaye, 2012). In addition, in 2014, 25% of Latino and Black households were multigenerational (Cohn & Passel, 2016). Capacities important to parenting quality, specifically attachment and EF, are still developing in teens and can affect their ability to effectively parent a young child. This may help explain why the teen's mother is often identified as an important source of support for teen mothers and their children (Beers & Hollo, 2009; Hudson et al., 2016; Spieker & Bensley, 1994). Evidence continues to emerge of intergenerational transmission of parenting behaviors and quality; most compelling is the influence of grandmothers (Kretchmar & Jacobvitz, 2002; Madden et al., 2015). Greater social support and more positive grandmother relationships have been associated with greater parenting competence and higher levels of nurturing behaviors among teen mothers (Oberlander, Black, & Starr, 2007; Sellers, Black, Boris, Oberlander, & Myers, 2011).

This report is a description of the protocol of a current study of the relationship of teen mothers’ (TM) and their mothers’ (grandmothers of the child‐ GM) or grandmothers’ (great‐grandmothers of the child‐ GGM) attachment and executive function capacities to the development of their young children. The research questions are:

1. What is the relationship between TMs’ and GMs’/GGMs’ attachment styles?
2. Does attachment style of TM or GM/GGM differ for young children with normal development than for those with risk for developmental delay?
3. What is the relationship between TMs’ and GMs’/GGMs’ level of EF?
4. Is there a difference in EF among TMs or GMs/GGMs based on the developmental status of their young child (normal vs. risk for developmental delay)?

While the cross‐sectional nature of the study does not allow for causality to be determined, study findings will add to knowledge of essential components of effective parenting in multi‐generational families with a parenting adolescent and their relationship to child development.

## 3 METHODS

### 3.1 Sample

Teen mothers, their biological, co‐habiting mothers or grandmothers, and their young children 1 to 3 years of age are being recruited from two teen‐tot clinics associated with an urban teaching facility in Washington, DC. The mean age of teen mothers served at this clinic is 17 years old. They are predominantly Black (94%) or Latina (5%), 90% are Medicaid‐insured, and approximately 85% are enrolled in an academic program, for example, high school, GED program, college, or vocational training.

#### 3.1.1 Inclusion and exclusion criteria

Inclusion criteria for TMs are age 15–19 years (inclusive), the biological mother of a child 1–3 years of age (inclusive), able to speak and read English at least at the 5th grade level (assessed by having completed at least an 8th grade education and ability to communicate with the recruiter), and living with their biological mother (GM of the child) or grandmother (GGM of the child) and the target child. GM/GGM inclusion criteria are being the biological mother or grandmother of the TM, ability to speak and read English at least at the 5th grade level, and living with the TM and the target child. Families of children with congenital abnormalities or chronic conditions known to affect development (e.g., Down Syndrome, cerebral palsy, fetal alcohol syndrome) are excluded because it would not be possible to determine if delays are linked to parenting factors or the underlying medical condition.

### 3.2 Sample size and power analysis

Based on the large association between maternal avoidant attachment and developmental outcomes of early childhood (*r* = −.84), with power = .80 and α = .05, a sample size of 22 in each group of TMs and GMs/GGMs (groups of those whose children have normal development and those whose children have risk for developmental delay) would enable detection of a significant relationship between avoidant attachment style and child developmental status. This effect size was used in power analysis, as no published reports were found on effect size for associations between EF in mothers or grandmothers and developmental status in their young children. A sample size of 50 dyads was chosen as a feasible goal to carry out this exploratory work.

### 3.3 Measures

#### 3.3.1 Attachment Style Questionnaire (ASQ)

This self‐report measure of 40 items rated on a 6‐point scale measures discomfort with closeness, need for approval, preoccupation with relations, viewing relationships as secondary (to achievement), and lack of confidence. The questionnaire yields two dimensions (avoidant and anxious attachment styles), each on a continuous scale. Only avoidant attachment will be analyzed in this study. A higher score indicates a more avoidant attachment style. The scale has internal consistency reliability of α = .85 in a sample of low‐income African American mothers (Alhusen et al., 2013). There is also evidence of convergent, discriminant, and predictive validity (Ravitz, Maunder, Hunter, Sthankiya, & Lancee, 2010).

#### 3.3.2 The Cambridge Neuropsychological Test Automated Battery (CANTAB)

This is a widely used computerized battery for ages 4 to 90 years (Smith, Need, Cirulli, Chiba‐Falek, & Attix, 2013). The battery contains nonverbal subtests, consisting of geometric shapes and designs, measuring the major domains of EF. A training test and three subtests (each looking at a domain of EF: working memory, inhibitory control, cognitive flexibility) are administered on a touch screen tablet, with standardized instructions from the test administration guide. Each test begins at a simple level, so that all participants score above the starting levels. As participants are successful at one level, they continue to more difficult versions of the test, which avoids ceiling effects. Scores vary by subtest but may range from −100 to ∞.

Alpha reliabilities in a pediatric sample were .73 to .95 (Luciana, 2003). CANTAB's construct validity is well supported in wide use across diverse groups of participants. Scoring across subtests follows developmental trajectories of executive function across age/developmental groups and discriminates between normal controls and multiple patient samples (Luciana, 2003; Smith et al., 2013). A composite score will be created to assess overall EF.

#### 3.3.3 Ages and Stages Questionnaire, 3rd edition (ages & stages‐3)

This is a standardized 30‐item maternal report of child development in communication, gross motor, fine motor, problem‐solving, and personal‐social skills. Scores range from 0 to 60, with age‐normed cut‐off scores that indicate normal, borderline, or delayed development. This tool has strong sensitivity (.86) and specificity (.85; Squires, Bricker, Twombly, & Potter, 2009). Validity of the ASQ‐3 is demonstrated in urban, ethnic minority, low‐ income populations (Guevara et al., 2013; San Antonio, Fenick, Shabanova, Leventhal, & Weitzman, 2014).

#### 3.3.4 Center for Epidemiologic Studies short depression scale (CESD‐10)

Depression is a common indicator of maternal distress that has demonstrated impact on child development (Huang, Costeines, Kaufman, & Ayala, 2014). Children of mothers with depressive symptoms have higher odds of low vocabulary, socio‐emotional issues, and negative effects on cognitive development (Azak, 2012; Kiernan & Huerta, 2008; Letourneau, Tramonte, & Willms, 2013). In particular, higher levels of maternal depression among teen mothers are associated with greater developmental delays in their toddlers (Huang et al., 2014). Depressive symptoms also have a negative impact on parenting qualities including maternal‐child interaction, maternal feelings of attachment to the child, as well as maternal cognitions (Logsdon, Wisner, & Pinto‐Foltz, 2006; Mason, Briggs, & Silver, 2011; Stein et al., 2012). Depression is known to interact with maternal parenting capacities, parenting, and child development. Measuring depression therefore provides an opportunity to control for confounding effects.

CESD‐10 is a self‐ report scale used to identify depressive symptoms. Each of 10 symptoms is rated on a four‐point scale. It has good reliability, with Cronbach's α = .86 (Miller, Anton, & Townson, 2008), sensitivity (.91) and specificity (.92; Zhang et al., 2012). This tool has been used for research in the general population (Radloff, 1977) and to identify depressed mood in pregnant adolescents (Salazar‐Pousada, Arroyo, Hidalgo, Perez‐Lopez, & Chedraui, 2010).

#### 3.3.5 Demographics

Data on age, race/ethnicity, relationship status, employment status, education completed, and average time spent per day with the young child are requested via questionnaire from each participant. GMs/GGMs are asked their total annual household income.

### 3.4 Recruitment and data collection

The study protocol was reviewed and approved by the recruitment institution's IRB. Recruitment flyers are posted in and around the teen‐tot clinic and given to clinic staff to share with eligible participants. A member of the research team reviews clinic appointment schedules weekly with the clinic social workers to identify eligible teens with appointments scheduled for themselves or their child(ren). When an eligible participant has an appointment, the researcher meets with the TM that day to inform them about the study and confirm eligibility. If the TM expresses interest in participation, contact information is exchanged, and the TM/GM dyad is scheduled for a study appointment at a mutually agreed upon time and location. Most study visits are conducted in participants’ homes or elsewhere in the community. At the study visit, written consent and/or assent is obtained, as indicated.

Participants have the option to complete questionnaires (demographics, ASQ, Ages & Stages‐3) independently or have each item read aloud and their responses recorded. Data are collected simultaneously from TMs and GMs in the same room; while one participant completes the CANTAB, the other completes the questionnaires. After completion, the participants complete the opposite measure(s). The Ages & Stages‐3 questionnaire is only completed by one participant, most often the TM. Data are reviewed at the end of the study visit to ensure there are no missing data. On average, study visits last 60–90 min. TMs and GMs/GGMs each receive a $25 gift card for completing research measures.

### 3.5 Analysis plan

Data will be analyzed using Stata Statistical Software: Release 14. Descriptive statistics will be used to summarize sample characteristics. Frequencies and percentages will be used to summarize categorical data. Means and standard deviations will be used to summarize continuous data. A mean value will be inserted if there are any missing data on the ASQ, CESD‐10, or Ages & Stages‐3.

To examine the relationships among teen mothers’ (TM) and their mothers’ (GM) or grandmothers’ (GGM) attachment style and EF (research questions 1 and 3), the intraclass Pearson product‐moment correlation coefficient will be used, or intraclass Spearman's rank order correction if attachment style or EF scores appear to deviate from a normal distribution. Strength and significance of correlations will be assessed.

At the time of analysis, the sample will be divided into two groups based on the developmental status of the young child: those with normal development and those with risk for developmental delay. Categorization will be based on results of the Ages & Stages‐3. Any child with a score in the “borderline” or “delay” range in one or more domain will be placed in the risk for delay group. In order to examine the influence of TM or GM/GGM attachment style and EF (composite scores) on child developmental status, *t*‐tests will be used to detect differences (research questions 2 and 4). This method was chosen given the smaller sample size. Based on findings of the relationships of attachment style and EF across the two generations, the combined effects of TM and GM/GGM on child's developmental status will be considered for analysis. Demographics are not planned as covariates in analysis, due to heterogeneity of the sample and limitations in power with regard to sample size. However, *t*‐tests may be used to look at demographic differences in TMs and GMs/GGMs that may influence the young child's development, for example, time spent per day with the child and education completed.

## 4 DISCUSSION

This study will be the first known exploration of attachment and executive function capacities across generations of parents and its relationship to development of young children. This study has the potential to inform future three‐generation research and interventions tailored to the needs of teen mothers to improve outcomes and reduce the risk of health disparities and social disadvantage.

To date, over 15 months of recruitment, over half of the target sample for data collection has been enrolled (34 dyads). The racial/ethnic make‐up is similar to the recruitment site, 95.5% Black/African American, 2.9% Latina, and 1.5% other. The mean age of TMs is 18 years old. Twenty‐nine grandmothers (age range: 36 to 56 years old) and 5 great‐grandmothers (age range: 62 to 68 years old) have participated. Most of the data collection has taken place in family homes. Other sites for data collection in the participants’ communities have included a WIC clinic, an eatery, and a study team member's car.

One of the biggest challenges has been enrolling a sufficiently large sample to enable between‐group differences to be detected. Although the clinic serves a large number of teen mothers, the specificity of inclusion criteria narrows the sample pool. To address this, several modifications were made to the inclusion criteria. First, age range for TMs was expanded from 17–19 years to 15–19 years, which is more in line with how pregnant and parenting teens are described and reported in the literature. A second revision to the inclusion criteria was to include biological, co‐residing grandmothers of the TM (GGM). The original study protocol required the participation of biological, co‐residing mothers of the TM (GM), but the number of grandparents raising children has grown; as of 2012, nearly 3 million grandparents were raising their grandchildren (Cohn & Passel, 2016; United States Census Bureau, 2014). Expanding this criterion better reflects the varied constellations of these families. Last, a satellite clinic in the same city was added as a second recruitment site. This site is part of the same teen‐tot program and serves a similar population of families.

Another challenge has been transience of the study population. Their contact information, living arrangements, availability, and responsiveness to contact are subject to change. Consistent follow‐up and flexibility in scheduling has been important, from the point of initial contact to introduce the study to the completion of data collection at the study appointment. However, follow‐up voice calls and text messaging by the study team have helped communication with participants. Participants have been equally responsive to both forms of communication. Several services offer free calling and texting using a variety of devices and have the option for a unique telephone number. Other strategies to support data collection include availability of evening and weekend hours to better accommodate family school and work schedules, reminder texts or calls the day before or morning of a scheduled study appointment, and flexibility in scheduling. For example, in response to follow‐up calls, participants have asked if the study appointment can be completed “now or later on today.”

Use of the CANTAB has brought certain challenges. A strength of this measure is that literacy level and learning barriers do not confound the data, as might happen with a paper and pencil measure. However, some participants find the computerized administration challenging. For example, the subtests can be repetitive, requiring participants to complete numerous trials within a task. Participants have been visually exasperated and even asked, “When is this over?” In these instances, participants are advised on the number of remaining tasks, for example, there is one more task after this one, or this task asks you to do this same thing four more times. The battery can also be frustrating for some participants, as the instructions simply indicate the task they are to do, without offering strategies on how to be successful in completing a task or providing insight on why a response is incorrect. One TM participant commented in response to repeated messages of an incorrect answer, “Wait, is it a pattern? I don't get it.”

Reassurance that other participants as well as the data collector have expressed frustration while completing tasks is also helpful. One GM participant offered guidance to her daughter by suggesting, “Take your time. It's patience. It's looking at how your brain works… a psychological test. Take your time. I got frustrated with it myself.” In previous studies, the computerized format has been found to be “interesting and motivating” for young people (Luciana, 2003), but thus far, in our sample, GMs and GGMs seem to have found the CANTAB more interesting than have teen mothers. One GM asked if the battery was available for private use, like a mobile app to play on her phone. Others have described it as “being like a game” and helping your mind and concentration. Despite the varied responses to the CANTAB, all participants to date have completed the battery.

### 4.1 Limitations

The use of self‐report measures may introduce reporting bias. The option for each participant to complete the measures with privacy and the assurance of confidentiality should minimize this bias, and multiple data collection methods (e.g., questionnaires and computerized battery) and multiple informants also may mitigate this limitation. The developmental screening survey, administered at a single time point, does not confirm a developmental delay. In future studies, more robust developmental assessments over time are warranted. Use of a convenience sample from a population already engaged in the health care system limits generalizability of the findings. Study participants are all women, which does not capture the importance and impact of fathers on child development, and inclusion of fathers is recommended for future studies. However, women are often identified as the primary parents. The power analysis for this study was guided by a robust effect size found in a single study. While we hope to find meaningful effects in this fairly small sample, this poses a limitation.

## 5 CONCLUSION

Despite its limitations, in this small study we are focusing on vulnerable families at high risk for health disparities and social disadvantage. We are using a novel approach to understanding two of the essential components of effective parenting in multigenerational families and parental/familial capacities that influence early child development, which will be useful in informing future studies.
